# Effect of addition of bioactive glass to resin modified glass ionomer cement on enamel demineralization under orthodontic brackets

**DOI:** 10.4317/jced.55576

**Published:** 2019-06-01

**Authors:** Maryam Shirazi, Mohamadali Tamadon, Mozhgan Izadi

**Affiliations:** 1 Assistant Professor of Orthodontics, Dental Caries Prevention Research Center, Qazvin University of Medical Sciences, Qazvin, Iran; 2 General Dentist, Student Research Committee, Qazvin University of Medical Sciences, Qazvin, Iran; 3 Assistant Professor, Dental Material Research Center, Department of Periodontics, Dental Research Institute, Isfahan University of Medical Sciences, Isfahan, Iran

## Abstract

**Background:**

Enamel demineralization and white spot lesions around orthodontic brackets is a common problem associated with fixed orthodontic treatment. Glass ionomer cements (GICs) are commonly used for bracket bonding and recently, bioactive glass (BAG) was added to GICs to enhance deposition of calcium phosphate in adjacent dental structure. This study sought to assess the effect of addition of BAG particles to resin modified GIC (RMGIC) on enamel demineralization under orthodontic brackets.

**Material and Methods:**

In this *in vitro*, experimental study, 60 sound human premolars were immersed in distilled water at 6°C. The teeth were randomly divided into three groups. Using a sticker, a window measuring 7x7 mm was created on the buccal surface. The remaining surfaces were coated with nail varnish twice with a 3-hour interval. After 24 hours, the stickers were removed. Brackets were bonded to tooth surfaces using Transbond XT (control), Fuji II LC RMGIC and Fuji II LC containing 30% BAG particles. To induce acid attacks, the teeth were immersed in demineralizing solution for 6 hours. For pH cycling, the teeth were then immersed in remineralizing solution for 18 hours. This process was repeated for 21 days. Next, the teeth were longitudinally sectioned into two halves and the depth of demineralization was measured from the deepest point to the surface under a polarized light microscope. Data were analyzed using one-way ANOVA, and pairwise comparisons were carried out using Tukey’s test.

**Results:**

The mean depth of demineralization was 73.8±22.29, 118.08±29.42 and 182.98±20.69 µm in the BAG, RMGIC and Transbond XT groups, respectively. One-way ANOVA showed significant differences in terms of depth of demineralization among the three groups (*P*=0.0001). Tukey’s test revealed significant differences in depth of demineralization.

**Conclusions:**

Addition of BAG to RMGIC can significantly decrease the depth of enamel demineralization under orthodontic brackets *in vitro*.

** Key words:**Remineralization, demineralization, orthodontic brackets, bioactive glass, resin modified glass ionomer cement.

## Introduction

Enamel demineralization and white spot lesions around orthodontic brackets is a common problem associated with fixed orthodontic treatment (1). Bands, brackets and other fixed orthodontic appliances such as elastics, springs and traction hooks complicate oral hygiene and enhance plaque accumulation (1).

Incidence of enamel demineralization in orthodontic patients is much higher than that in control individuals (2). On the other hand, prevalence of enamel demineralization in orthodontic patients ranges from 2-96% (2).

Composite resins are commonly used by orthodontists for bracket bonding due to easy use and decreased working time (3). Glass ionomer cements (GICs) were first suggested by Wilson and Kent (1972) as the material of choice for esthetic restoration of anterior teeth (3). These cements are biocompatible with enamel and dentin and have cariostatic effects. The fluoride ions in their composition initiate remineralization (4). These cements are biocompatible and release fluoride. Therefore, they are the material of choice for restoration of teeth in areas which are hard to isolate (such as second molars, surgically exposed teeth and lingual surface of mandibular teeth) (5). However, the bond strength of these cements is clinically low; thus, they are not routinely recommended for orthodontic bracket bonding (6).

Resin modified GICs (RMGICs) were later introduced to enhance the bond strength (4). RMGICs are composed of components of GICs (fluoroaluminosilicate glass and polyacrylic acid) and composite resins (photo- or chemical initiators and methacrylate monomers) (7). Due to the presence of resin component, these cements have higher bond strength to the enamel surface (4). On the other hand, this group of cements, compared to conventional GICs, have lower technical sensitivity and more favorable physical and mechanical properties (8). The fluoride release potential of these cements is similar to that of conventional cements (9).

Bioactive glasses (BAGs) include surface reactive glasses, which enable bone minerals to form chemical bonds (10). The composition of BAGs includes calcium oxide, sodium oxide, phosphorus oxide and silicon oxide in specific volume percentages (11). They all form compounds with unique surface reactivity (12). BAGs can also be incorporated into the composition of GICs to exert antibacterial effects (13). BAG was introduced in 1971 for use in dental implants. It has excellent biological activity and biocompatibility with bone and soft tissue. Recently, BAG was used as a therapeutic substance in areas at high risk of microbial infections. It has a wide-range of antibacterial activity against supragingival and subgingival bacteria. Thus, antibacterial activity of BAG has been the topic of many studies in the field of dental materials (14).

This study aimed to assess the effect of addition of BAG particles to RMGIC on enamel demineralization under orthodontic brackets.

-Sample size

**Figure 1:**
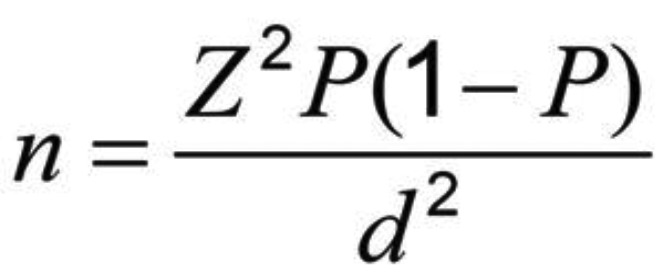
Formula.

Considering the number of samples in the same research (29), and taking into account the95% accuracy of the test, and using the following formula, the number of samples required for each of the three groups in the study was estimated 20 (Total 60 samples), (Fig. 1).

## Material and Methods

This study approved by ethical committee of Qazvin University of Medical Sciences with ethical number of IR.QUMS.REC.1395.15. There is no conflict with ethical considerations. This *in vitro*, experimental study was performed on 60 sound, extracted human premolar teeth, which were free from cracks, restorations or caries. The teeth were inspected under a microscope to ensure that they met our inclusion criteria. The teeth were immersed in 0.1% thymol solution for one week and stored in distilled water at 6°C until the experiment (15). Tissue residues and calculus were removed using a periodontal curette. The buccal surface of the teeth was cleaned with pumice paste and rubber cap with low speed hand piece for 10 seconds. The teeth were then randomly divided into three groups (n=20). All teeth were prepared by the same experienced operator within two consecutive days.

The study groups were as follows:

Group one or control group: Transbond XT (3M, St. Paul, MN, USA)

Group 2: Fuji II LC RMGI (GC Corp., Tokyo, Japan)

Group 3: Fuji II LC (GC Corp., Tokyo, Japan) containing 30% BAG particles 

Preparation of samples.

After cleaning of the buccal surface, a sticker measuring 7x7 mm was placed on the buccal surface of the teeth in all groups. The remaining surfaces were coated with nail varnish twice with a 3-hour interval. After 24 hours, the stickers were removed and the residual adhesive was cleaned using a cotton pellet dipped in alcohol. To ensure than the surface was clean, the area was inspected under a stereomicroscope. Care was taken to bond the bracket to the center of the created window, and the surrounding area was used as the control area.

In group 1, surfaces were etched with 37% phosphoric acid (Ultradent) for 30 seconds, rinsed for 10 seconds and air-dried with gentle air spray. Next, Transbond XT primer (3M, St. Paul, MN, USA) was applied to the tooth surface using an applicator and light-cured for 10 seconds using a LED light curing unit (LE Demetron, SDS Kerr, USA) with 470 nm wavelength and 1100 mW/cm2 light intensity. Composite resin was then applied to the bracket base (Edgewise, Standard, metal, 0.22, American Orthodontics) and the bracket was positioned at the center of the surface using a bracket positioner. Excess material was removed by a dental explorer and light curing was performed for 40 seconds (10 seconds from each side of bracket).

In group 2, the buccal surface of the teeth was cleaned and then etched with 37% phosphoric acid (Ultradent) for 30 seconds, rinsed with water and air spray for 10 seconds and dried with gentle air spray. According to the manufacturer’s instructions, one scoop of RMGIC powder (Fuji II LC, GC Corporation, Japan) was mixed with two drops of liquid. For this purpose, the powder was divided into two portions and each portion was separately mixed with the liquid for 10 seconds using a plastic spatula to obtain a glossy consistency. The cement was then applied to the bracket base and the bracket was placed at the center of the tooth surface using bracket positioner. Excess cement was removed by a dental explorer and light curing was performed for 40 seconds (10 seconds from each side of bracket).

In group 3, 30v% of BAG was added to RMGIC powder and as in group 2, brackets were bonded to teeth. Next, the samples were immersed in distilled water and incubated at 37°C for 24 hours.

To simulate the changes in oral environment after food consumption, pH cycling was performed. For this purpose and to simulate acid attacks, the teeth were exposed to demineralizing solution for 6 hours and were then exposed to the remineralizing solution for 18 hours. This process was repeated for 21 days. The volume of each solution for each sample was considered to be 10 mL. All these procedures were performed in an incubator at 37°C and the solutions were refreshed once a week.

P-reparation of solutions.

To prepare the demineralizing solution with the formulation of 2.2 mM CaCl2, 50 M CH3COOH and 2.2 mM KH2PO4, 35.78 mL of 1M citric acid, 14.22 mL of 1M sodium acetate, 0.0022 M (0.3 g) of potassium dihydrogen phosphate and 0.0022 M (0.25 g) calcium chloride were poured in a 1 L container and the volume was reached to 1 L using deionized water. The obtained solution had a pH of 4.3.

To prepare the remineralizing solution with the formulation of 1.5 mM CaCl2, 150 mM KCl and 0.9 mM KH2PO4, the amount of material (molecular mass) was calculated and 0.165 g of calcium chloride, 11.1 g of potassium chloride and 4.76 g of potassium dihydrogen phosphate were added to a 1 L container and its volume was reached to 1 L using deionized water. The pH of this solution was adjusted at 7.

-Sample preparation for microscopic assessment.

All teeth were first sectioned along their longitudinal axis into two halves with a Discoplan-TS (Struers) sectioning machine. The teeth were wet-polished with carborundum powder (400 and 800 grit). Using synthetic thermoplastic resin (Eukitt), the polished surface was fixed on a slide and the diameter of samples was adjusted at 30-100 µm using the above-mentioned polishing machine.

-Assessment under polarized light microscope.

**Figure 2:**
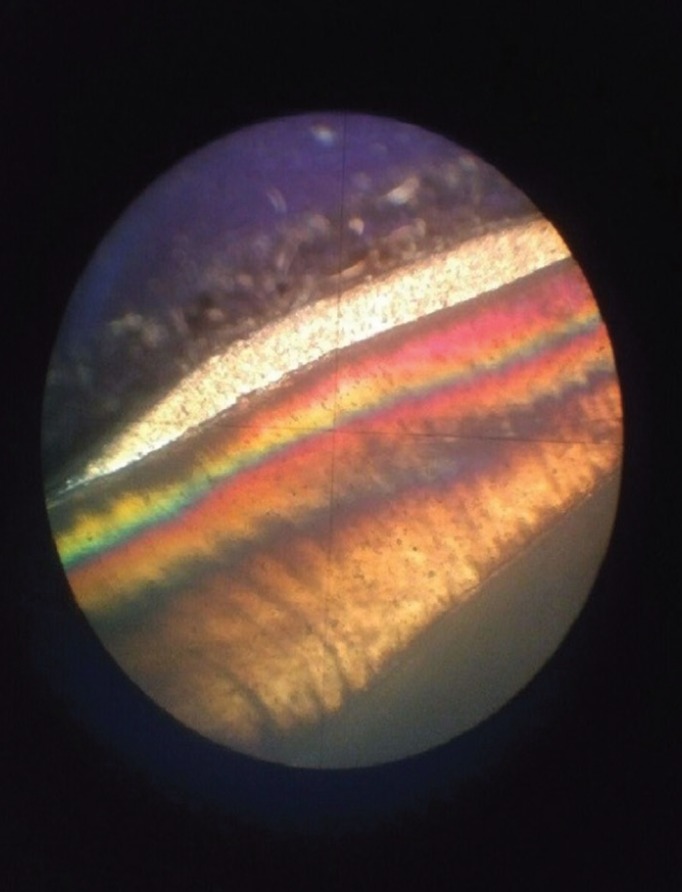
Demineralized lesion with polarized light microscope.

The sections were stored in distilled water and were then evaluated under a magnifier at x10 and x5 magnification (Fig. 2). Depth of lesion was measured from the deepest point to the surface under polarized light microscope (BH2; Olympus).

According to the respective table for the microscope, each degree of the graded lens at x50 magnification specified 20 µm of the specimen. To measure the depth of lesion, the code of each slide was covered with a sticker and depth of lesions was measured by two trained observers who were not aware of the group allocation of specime. The agreement between the measurements of two observers was evaluated by the Kappa coefficient, which resulted in a value of 0.8. Due to an acceptable agreement between the two observers, the mean depths recorded by the two observers were analyzed.

The mean, standard deviation, minimum and maximum values of demineralization depth of enamel lesions under orthodontic brackets were calculated and reported in the three groups and compared using one-way ANOVA. Pairwise comparisons were carried out using Tukey’s test.

## Results

The mean (± standard deviation) depth of demineralization was 73.8±22.29 µm in BAG, 118.08±29.42 µm in RMGIC and 182.98±20.69 µm in Transbond XT group ( Table 1). One-way ANOVA showed a significant difference in depth of demineralization among the three groups (*P*=0.0001). Tukey’s test showed significant differences in demineralization depth between BAG and RMGIC (*P*<0.0001), BAG and Transbond XT (*P*<0.0001) and RMGIC and Transbond XT (*P*<0.0001) groups.

**Table 1:**
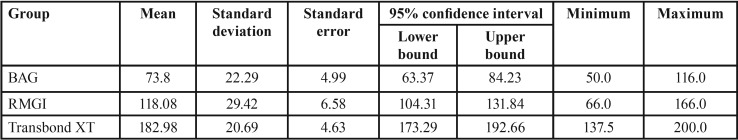
Mean and standard deviation of demineralization depth under brackets following pH cycling in the three groups of BAG, RMGIC and Transbond XT.

## Discussion

The results of this study showed significant differences in terms of demineralization depth in the three groups. Also, pairwise comparisons revealed significant differences in this respect between BAG and RMGIC, BAG and Transbond XT and RMGIC and Transbond XT groups. BAGs have been recently used for many dental purposes. It has been documented that BAG can induce the deposition of calcium phosphate. The current results showed that RMGIC containing BAG had the greatest ability to prevent demineralization under orthodontic brackets. Adhesives containing BAG can release calcium ions into their surrounding solutions (16).

Since BAG particles are easily transferred in the clinical environment, a carrier or matrix such as GIC is required to enhance their clinical application. Thus, in the current study, BAG particles were added to RMGIC to determine their efficacy for prevention of enamel demineralization around orthodontic brackets.

Mitchell *et al.*, (2006) in their *in vitro* study showed that GIC containing BAG prevented the growth of cariogenic bacteria and these cariostatic effects may explain the ability of BAG to prevent demineralization and white spot lesions (17). Following immersion of GICs containing BAG in aqueous solutions, bioactive materials release ions into the surrounding environment and it seems that BAG particles have large surfaces and high porosities and therefore, have high capability to release ions (18).

The ability of BAG to release ions is due to the weak ionic bonds and easier release of calcium into the surrounding environment compared to the tooth surface. Sodium ions present in the composition of BAG are replaced with hydrogen ions and the pH of the solution increases as such (19). Consequently, calcium and phosphate ions are transferred to the surface and a layer rich in calcium phosphate is formed on the tooth surface (19). This ionic reservoir can protect the enamel against cariogenic challenge, inhibit demineralization and increase remineralization (19). After immersion of calcium sodium phosphosilicate compounds in the saliva analogues or body fluids, Na+ ions are immediately exchanged with hydrogen cations (H+ or H3O+) within one minute (20) and this fast exchange of ions results in release of calcium (Ca2+) and phosphate (PO43-) from the structure of particles. The temporary and local increase in pH occurs and leads to release of calcium and phosphate from the particles and also from the saliva for the formation of an amorphous layer of calcium phosphate (CaO-P2O5) on the tooth surface and in the demineralized dentin. As the reactions and deposition of Ca-P compounds continue, this layer is crystalized within the hydroxyapatite, which is chemically and structurally the same as biologic apatite (20). On the other hand, combination of residual calcium sodium phosphosilicate and the hydroxyapatite layer causes remineralization and physical obstruction of dentinal tubules. Chemical reactions are also initiated by calcium sodium phosphosilicate to enhance the formation of hydroxyapatite and may be useful for treatment of demineralization of tooth structure. Furthermore, they prevent further demineralization and enhance remineralization.

Due to the presence of fluoride in the composition of RMGIC and its constant release, fluoride may participate in the mineral composition of tooth structure and result in formation of insoluble fluorapatite (16). In the current study, RMGIC containing BAG had greater capability in decreasing the depth of enamel demineralization lesions under orthodontic brackets due to its ability to release ions and having higher biological activity.

Brown *et al.* (2011) assessed the ion release potential of BAG and showed that BAG decreased the speed of enamel demineralization by increasing the pH around the tooth/orthodontic bracket interface or releasing a large volume of calcium ions into the solution (16). These observations were confirmed in our study. Manfred *et al.* (2013) evaluated changes in enamel microhardness adjacent to orthodontic brackets following the application of bonding agents containing different BAG products and conventional adhesive resins under simulated cariogenic conditions and revealed that the bonding agent containing BAG significantly prevented enamel softening around brackets compared to the conventional bonding agents (21). In their study, similar to ours, Transbond XT was used as the control group and the 14-day cariogenic challenge was performed according to the modified pH cycling protocol. However, the ability to prevent demineralization in their study was assessed by the Knoop hardness test while a polarized microscope was used for this purpose in the current study.

Hassanein and El-Brokossy (2006) assessed remineralization by BAG in the enamel and dentin with artificial caries using Raman spectroscopy and revealed that BAG was capable of remineralization of artificial caries in the enamel and dentin and these results were confirmed by our current findings (22). Prabkakar *et al.* (2010) indicated deposition of minerals at the restoration-dentin interface and into deep dentinal tubules especially in use of RMGIC containing BAG and stated that this mixture seemed to be able to remineralize dentin (23). Palaniswamy *et al.* (2016) assessed the remineralizing potential of BAG for primary enamel lesions and showed that it was effective for prevention of demineralization (24). It seems that addition of BAG to RMGIC provides a rich source of calcium, phosphate and fluoride ions to prevent enamel demineralization around orthodontic brackets. Therefore, some studies evaluated the efficacy of regular use of calcium- and fluoride-containing solutions to increase the duration of ion release.

The current results showed that RMGIC significantly decreased enamel demineralization depth under orthodontic brackets compared to the control group. It has been documented that GICs containing fluoride have high cariostatic potential (25). This partly depends on the role of fluoride in increasing the deposition of calcium phosphate and subsequent enamel remineralization (26).

Optimal properties of RMGIC include insignificant solubility in oral fluids, the ability to bond to enamel in the oral environment, high shear bond strength, suitable applicability and fluoride release potential. Thus, it is believed to be suitable for prevention of caries around orthodontic brackets (27). In the conventional GICs, due to less water sorption, BAG particles form limited reactions with polyacrylic acid, and BAG particles participate in small superficial reactions (10). This is the main reason explaining why RMGICs react more with BAG (23).

In presence of fluoride, saliva and its analogues are often saturated with fluorapatite in most pH values while super-saturation for hydroxyapatite occurs in the pH range of 5.6-5.8. Moreover, in presence of less amounts of fluoride in the saliva or dental plaque fluid, hydroxyapatite may be dissolved at its critical pH but dissemination of mineral ions can cause re-deposition of fluorapatite or fluorohydroxyapatite. This mechanism prevents mineral loss and provides extra support for mineral crystals via increasing the fluoride content of the outer layers for the formation of apatite crystals. Enamel and dentin remineralization tests have shown that fluoride increases the uptake of minerals during the process of remineralization (28).

Wilson and Donly (2001) assessed prevention of enamel demineralization around orthodontic brackets by use of different materials and indicated that RMGIC and composite resin containing fluoride decreased enamel demineralization compared to the use of composite resin without fluoride (29).

Crystalline structures in the enamel mainly contain calcium and phosphorus and a small percentage of sodium, magnesium and carbonate. Also, enamel minerals are present in the form of biologic apatites, and occurrence of demineralization results in mineral loss especially calcium. Phosphate groups in hydroxyapatite can have a positive charge in acidic environments and result in dissolution of crystals. This mechanism has been used for simulation of caries *in vitro*. Buffering solutions with a pH less than 5 are often used for this purpose (30). This method, compared to the artificial dental plaque models, is less complex and requires less time than the use of acidic gels, which often require 12 to 15 months to cause demineralization.

## Conclusions

Addition of BAG particles to RMGIC significantly decreased the depth of enamel demineralization lesions under orthodontic brackets *in vitro*. Thus, by addition of BAG to RMGIC, enamel demineralization can be significantly prevented.
